# Effects of Acute Aerobic Exercise on Rats Serum Extracellular Vesicles Diameter, Concentration and Small RNAs Content

**DOI:** 10.3389/fphys.2018.00532

**Published:** 2018-05-24

**Authors:** Getúlio P. Oliveira, William F. Porto, Cintia C. Palu, Lydyane M. Pereira, Bernardo Petriz, Jeeser A. Almeida, Juliane Viana, Nezio N. A. Filho, Octavio L. Franco, Rinaldo W. Pereira

**Affiliations:** ^1^Programa de Pós-Graduação em Patologia Molecular, Universidade de Brasília, Brasília, Brazil; ^2^S-Inova Biotech, Pós-Graduação em Biotecnologia, Universidade Católica Dom Bosco, Campo Grande, Brazil; ^3^Bioinformatics, NSilico Life Science Ltd., Cork, Ireland; ^4^University College Cork, Cork, Ireland; ^5^Programa de Pós-Graduação em Ciências Genômicas e Biotecnologia, Universidade Católica de Brasília, Brasília, Brazil; ^6^Centro Universitário UDF, Brasília, Brazil; ^7^Programa de Pós-Graduação em Saúde e Desenvolvimento na Região Centro Oeste, Universidade Federal de Mato Grosso do Sul, Campo Grande, Brazil; ^8^Programa de Pós-Graduação em Educação Física, Universidade Católica de Brasília, Brasília, Brazil

**Keywords:** extracellular vesicles, small RNA (smallRNA), aerobic exercise, NextGene, edgeR

## Abstract

Physical exercise stimulates organs, mainly the skeletal muscle, to release a broad range of molecules, recently dubbed exerkines. Among them, RNAs, such as miRNAs, piRNAs, and tRNAs loaded in extracellular vesicles (EVs) have the potential to play a significant role in the way muscle and other organs communicate to translate exercise into health. Low, moderate and high intensity treadmill protocols were applied to rat groups, aiming to investigate the impact of exercise on serum EVs and their associated small RNA molecules. Transmission electron microscopy, resistive pulse sensing, and western blotting were used to investigate EVs morphology, size distribution, concentration and EVs marker proteins. Small RNA libraries from EVs RNA were sequenced. Exercise did not change EVs size, while increased EVs concentration. Twelve miRNAs were found differentially expressed after exercise: rno-miR-128-3p, 103-3p, 330-5p, 148a-3p, 191a-5p, 10b-5p, 93-5p, 25-3p, 142-5p, 3068-3p, 142-3p, and 410-3p. No piRNA was found differentially expressed, and one tRNA, trna8336, was found down-regulated after exercise. The differentially expressed miRNAs were predicted to target genes involved in the MAPK pathway. A single bout of exercise impacts EVs and their small RNA load, reinforcing the need for a more detailed investigation into EVs and their load as mediators of health-promoting exercise.

## Introduction

Physical activity is a non-pharmacological aid widely recognized as preventing and treating chronic metabolic disorders such as obesity, type 2 diabetes, cardiovascular disease, breast cancer, colon cancer, and ischemic stroke events (Admyre et al., 2007; Almeida et al., 2013). However, the molecules and the physiological processes behind such systemic benefits are still not completely understood. Exercise stimulates skeletal muscle to release a type of protein into the bloodstream; these molecules have been dubbed myokines (Almqvist et al., 2008) and, more recently, to include molecules other than proteins the term exerkines was suggested (Anderson and Ivanov, 2014). The importance of focused funding and research in understanding the molecular transducers behind the benefits of physical activity was recently recognized by the National Institute of Health, through a Common Fund Program (Aoi et al., 2013).

Since non-degraded circulating cell free small RNA was demonstrated in serum or plasma from cancer patients (Caby et al., 2005; Baggish et al., 2011) and also in muscle tissue injury (Chaturvedi et al., 2015), this drew attention as a biomarker and also as a potential molecular transducer in a plethora of pathological and physiological processes (Chen et al., 2008). The field of exercise science also took a keen interest. Circulating cell free RNAs, mainly microRNAs, have been investigated as biomarkers in different models of exercise intervention, such as aerobic training (Colombo et al., 2014), power training (Contarteze et al., 2008) and in competitive races (Da Sol Kim et al., 2015; D’Souza et al., 2017). It is not yet possible to call a set of circulating microRNAs that would be called more prevalent or related to specific aspects of exercise (Frühbeis et al., 2015).

As the number of studies investigating circulating cell free RNA increased, reproducibility was noticed to be an issue, mainly affected by technical decisions about sampling, RNA extraction and miRNA surveying (Gatti et al., 2005). Besides the attempt to establish methodological standards, the proposal to focus on circulating cell free RNA associated with extracellular vesicles (EVs) attempted to circumvent at least the biological complexity of circulating non-coding RNA (Gomes et al., 2015).

Extracellular vesicles include vesicles found in all fluids investigated so far, such as urine (Gomes et al., 2014), saliva (Goodarzi et al., 2016), milk (Guescini et al., 2015), seminal (Gupta and Knowlton, 2007) and amniotic fluids (Gutiérrez-Vázquez et al., 2013), serum (Hawley and Holloszy, 2009) and plasma (Hecksteden et al., 2016). These EVs may have their origin directly from cell membrane or from an endosomal pathway (Hu et al., 2010). The latter type are called exosomes. The demonstration that EVs harbor RNAs and that they can be shuttled among cells suggested that EVs may be new, and as yet unrecognized, cell-to-cell communication mediators (Ishimura et al., 2014). EVs and their associated RNA molecules had already been recognized as potential transducers of physical activity (Jarry et al., 2014). However, they are the last of the whole cell free circulating RNA repertoire to be investigated.

Here we present data from EVs and their global non-coding small RNA load investigated in serum from non-exercised and exercised rats. Our data indicate that exercise intensity affects EVs concentration and their non-coding small RNA load.

## Results

### Acute Aerobic Exercise Did Not Change Rat Serum EVs Diameter While Increases Serum EVs Concentration

**FIGURE 1:**
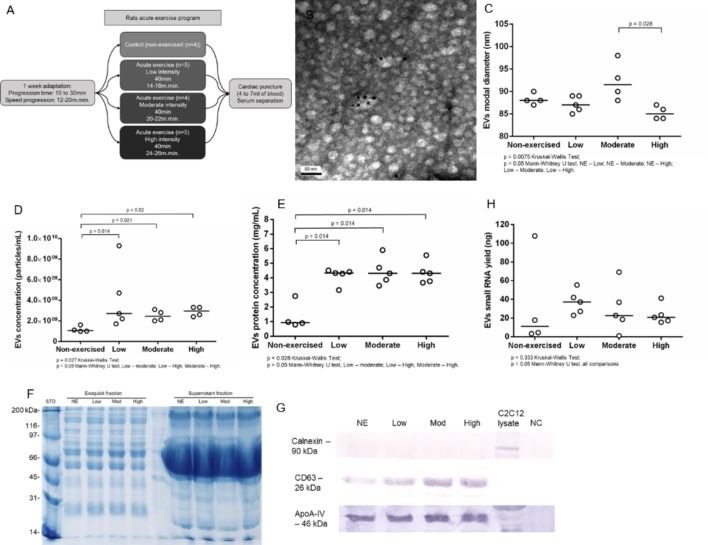
Acute aerobic exercise decreases rat serum EVs modal diameter and increases serum EVs concentration. **(A)** Workflow representing the acute exercise protocol used to provide aerobic exercise for Wistar rats running on a treadmill. **(B)** Immunoelectron micrograph of rat serum EVs purified by Exoquick. Black dots are 10 nm gold particle linked to the exosome marker CD63. **(C)** Tunable resistive pulse sensing (TRPS) analysis showed a slightly reduction in EVs modal diameter in high intensity exercised group compared to non-exercised (*p* = 0.057) and moderate intensity exercised groups (*p* = 0.028). The median groups for EVs modal diameter range from 88 nm in non-exercised, through 87 nm in low intensity, 91.5 nm in moderate intensity and 85 nm in high intensity exercised group **(D)**. The concentration of EVs purified from rat’s serum after exercise measured by TRPS. There is a statistical increase in median EVs concentration after exercise, which range from 1.1 × 10^9^ in non-exercised group to 3 × 10^9^ in low intensity exercised group (*p* = 0.014), 2.5 × 10^9^ in moderate intensity exercised group (*p* = 0.021) and 3 × 10^9^ in high intensity exercised group (*p* = 0.02). **(E)** The median concentration of rat serum EVs proteins. Proteins were purified from EVs and quantified. There is a statistical significance increase in EVs protein concentration median after exercise ranging from 0.935 mg.mL^-1^ in non-exercised group to 4.33 mg.mL^-1^ in low intensity exercised group (*p* = 0.014), 4.31 mg.mL^-1^ in moderate intensity exercised group (*p* = 0.014) and 4.31 mg.mL^-1^ in high intensity exercised group (*p* = 0.014). **(F)** Total rat serum protein profile after Exoquick purification analyzed by SDS-PAGE 12%. Exoquick fraction contains EVs proteins while supernatant fraction contains free proteins that were not precipitated by Exoquick. **(G)** Western blotting of EVs proteins shows an increase in the exosome marker CD63 while increasing the intensity of exercise. ApoA-IV protein levels remained stable regardless of exercise intensity. Calnexin was used to show the absence of endoplasmic reticulum proteins in purified EVs. **(H)** The concentration of EVs small RNAs from rat’s serum. Small RNAs were purified, quantified and characterized by 2100 Bioanalyzer (Agilent) using a small RNA chip. There is no statistical difference between group medians.

Aiming to understand the impact of acute aerobic exercise on the population of serum EVs, we submitted Wistar rats to acute exercise on a treadmill (**Figure 1A**). The EVs purification method commercialized as Exoquick^TM^ (Exosome Precipitation Solution, System Biosciences Inc., Mountain View, CA, United States) allowed us to purify EVs from rat serum as demonstrated by transmission electron microscopy (TEM) and immune electron microscopy. TEM analysis showed cup-shaped vesicles with size ranging from 40 to 200 nm, as expected for exosomes and microvesicles. Immuno electron microscopy showed that some vesicles were positive for exosomal membrane marker CD63, as demonstrated by 10 nm anti CD63 immunogold nanoparticles (**Figure 1B**). Tunable resistive pulse sensing (TRPS) analysis showed a slightly reduction in EVs modal diameter between non-exercised and high intensity exercised rats but this reduction was not statistically significant (*p* = 0.057). However, EVs modal diameter decreased in the high intensity exercised group compared to moderate intensity exercised group (*p* = 0.028). The groups median values for EVs modal diameter changed from 88 nm in non-exercised, through 87 nm in low intensity, 91.5 nm in moderate intensity and 85 nm in high intensity exercised group (**Figure 1C**). TRPS also showed that exercise significantly increased serum EVs concentration. The groups median values for serum EVs concentration was 1.1 × 10^9^ per mL in non-exercised group, 3 × 10^9^ for low intensity exercised group (*p* = 0.014), 2.5 × 10^9^ for moderate intensity exercised group (*p* = 0.021) and 3 × 10^9^ for high intensity exercised group (*p* = 0.02) (**Figure 1D**). Furthermore, exercise significantly increased serum EVs protein concentration, ranging from 0.935 mg.mL^-1^ in non-exercised group to 4.33 mg.mL^-1^ in low intensity exercised group (*p* = 0.014), 4.31 mg.mL^-1^ in moderate intensity exercised group (*p* = 0.014) and 4.31 mg.mL^-1^ in high intensity exercised group (*p* = 0.014) (**Figure 1E**). Overall, exercised groups behave differently than non-exercised group. Moreover, we didn’t find statistically significant differences regarding EVs concentration, EVs protein concentration and EVs small RNA yield when we compared between exercised groups (low to moderate intensity exercised groups, low to high intensity exercised groups, and moderate to high intensity exercised groups). Rat serum EVs protein profile was accessed by SDS-PAGE from the exoquick purified fraction and the exoquick supernatant. The Exoquick^TM^ fractions (EVs) visually show well-defined bands from 10 to 250 kDa. The supernatant fraction showed a pronounced band from 80 to 45 kDa, which we assumed were Albumin and IGG serum-enriched proteins. The Exoquick^TM^ precipitation method strongly reduce the concentration of these contaminants EVs proteins (**Figure 1F**). Western blotting showed that the concentration of CD63 positive vesicles increased from non-exercised group to high-intensity exercised group (**Figure 1G**), corroborating the TRPS data, which showed an increase in EVs concentration. Calnexin was used as a control for endoplasmic reticulum protein and was absent in EVs. Although is described that Exoquick^TM^ can purifiy both high density and low density lipoproteins (HDLs and LDLs), which contain miRNAs, we didn’t see an increase in ApoA-IV protein marker after exercise, indicating that the effects of exercise is more prevalent in CD63 positive vesicles than in ApoA-IV positive vesicles (**Figure 1G**). After performing biophysical and biochemical characterization from rat serum EVs, we purified and characterized EVs small RNA, aiming to conduct library construction and sequencing. Based on 150 μL of EVs suspension, we extracted small RNAs from all 18 samples (NE = 4, Low = 5, Moderate = 4, high = 5). The total yields of EVs small RNA between non-exercised and exercised groups were not statistically different (**Figure 1H**). All samples showed enrichment in RNAs between 15 and 40 bp, a common size relative to miRNA and other small RNAs (Supplementary Figure S1). In addition, we characterized larger RNAs with bioanalyzer and did not find peaks representing ribosomal subunits 18S and 28S.

### Acute Aerobic Exercise Changes Rat Serum EVs Small RNA Load

Aiming to understand the global small RNA expression in the population of rat serum EVs, we performed small RNA sequencing from non-exercised rats (*n* = 4) and low (*n* = 5), moderate (*n* = 4) and high (*n* = 5) intensity exercised rats. Raw read numbers obtained after sequencing, after filtering, trimming and alignment to *Rattus norvegicus* mature miRNA database, available at miRBase.org, are summarized in Supplementary Table S2. Overall, 24.92% of the total reads were considered mappable reads (after filtering and trimming). Reads that did not align to the previous database were aligned to the following one. The percentages of mappable reads aligned to the databases were: microRNAs 7.25%, piRNAs 16.63%, rRNA 1.77%, and tRNA 6.36% (Supplementary Figure S2).

**FIGURE 2:**
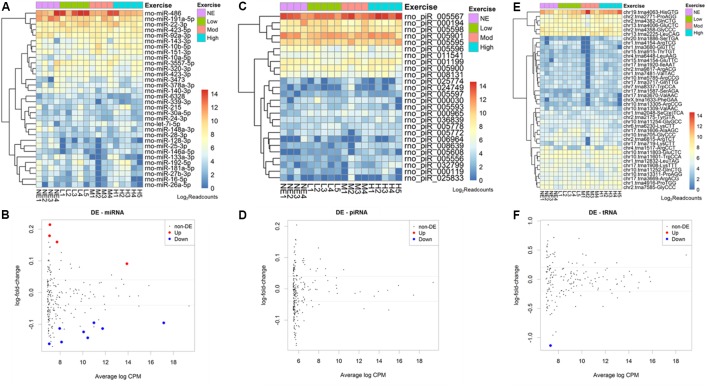
Acute aerobic exercise changes rat serum extracellular vesicle small RNA content. Heatmaps representing the most expressed serum EVs miRNAs **(A)** piRNAs **(C)** and tRNAs **(E)** purified from non-exercised and acute exercised rats. Figures **(B,D,F)** represent differentially expressed miRNAs **(B)**, piRNAs **(D)**, and tRNAs **(F)**. Each dot represents a small RNA average expression. Small RNAs that have a reduced expression after exercise are below black line and small RNAs that are increased after exercise are above black line. Red and blue dots represent small RNAs with statistically significant differences (FDR < 0.05). **(B)** After exercise, miRNAs rno-miR-330-5p, 10b-5p, 142-3p, and 410-3p were increased, while rno-miR-128-3p, 103-3p, 148a-3p, 191a-5p, 93-5p, 25-3p, 142-5p, and 3068-3p were decreased. **(D)** No piRNA was differentially expressed. **(F)** One tRNA, trna8336-AspGTC, was decreased after exercise in rat serum EVs.

From 765 mature miRNAs described in the *R. norvegicus* miRBase database, we found an average of 41 mature microRNAs (5.3%) aligned with at least 5 reads. The 10 most abundant mature miRNAs identified were: rno-miR-486, 191a-5p, 22-3p, 423-5p, 92a-3p, 143-3p, 10b-5p,151-3p, 10a-5p, 3557-5p (**Figure 2A**).

The number of reads for miRNA, piRNA, and tRNA in each sample was used in the edgeR analysis aiming to find small RNAs showing differential expression among non-exercised and exercised groups. We used a regression model that takes into account the treadmill velocity to search for differentially expressed EVs miRNA related to low, moderate and high intensity exercise. We found 12 miRNAs differentially expressed in serum EVs after exercise. Four miRNAs were upregulated after exercise: rno-miR-330-5p (FDR = 0.0284), 10b-5p (FDR = 0.0381), 142-3p (FDR = 0.0454) and 410-3p (FDR = 0.0461). Eight miRNAs were downregulated after exercise: Rno-miR-128-3p (FDR = 0.0238), 103-3p (FDR = 0.0284), 148a-3p (FDR = 0.0342), 191a-5p (FDR = 0.0342), 93-5p (FDR = 0.0381), 25-3p (FDR = 0.0381), 142-5p (FDR = 0.0414), 3068-3p (FDR = 0.0454) (**Figure 2B**).

Regarding piRNAs, from 36.279 rat’s piRNAs available in the piRNA bank, we found an average 133 piRNAs (0.3%) identified in rat serum EVs. From all of these, the 10 most enriched piRNAs were: rno-piR-5567, 194, 5598, 5901, 5595, 5596, 11541, 1199, 5900, 8131 (**Figure 2C**). We did not find any differentially expressed piRNAs between non-exercised and exercised rats (**Figure 2D**).

Furthermore, from 410 rat tRNAs available in the GtRNAdb database, we found an average 100 tRNAs (24%) identified in rat serum EVs. Among all tRNAs found, the 10 most enriched tRNAs were: tRNA4063, 2771, 4382, 4006, 4358, 2225, 11601, 12832, 11803, 11252 (**Figure 2E**). One tRNA, tRNA8336, was statistically differentially expressed after exercise, being downregulated (FDR = 0.0324) (**Figure 2F**).

### miRNAs – mRNAs Signaling Pathway Prediction Determines MAPK as a Main Target for Biological Function

**FIGURE 3:**
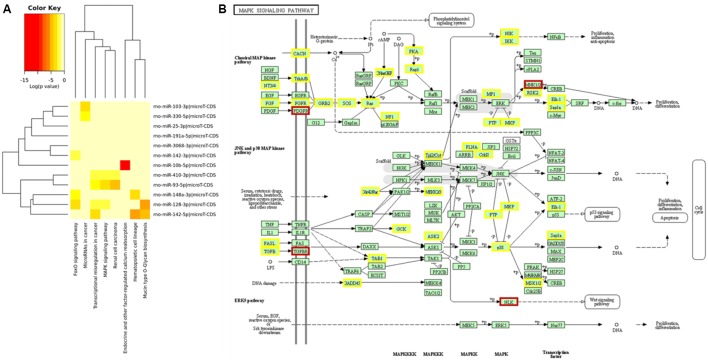
Main signaling pathways involved for 12 miRNAs differentially expressed after exercise from EVs purified from rat’s serum. Prediction for regulatory pathways. **(A)** 12 differentially expressed miRNAs after acute aerobic exercise regulate 8 signaling pathways. **(B)** MAPK signaling pathway was the pathway with more miRNAs regulating their genes. Genes regulated by one miRNA are highlighted in yellow, and genes regulated by two miRNAs are highlighted in brown. The most regulated genes from MAPK pathway were: platelet derived growth factor receptor beta (PDGFRA), a target for miR-93-5p, 410-3p and 128-3p; MAP kinase-interacting serine/threonine kinase 2 (MKNK2), a target for miR-93-5p and 128-3p; Nuclear factor of activated T-cells 3 (NFATE3), a target for miR-128-3p and 103-3p; transforming growth factor beta receptor 1 (TGFBR1), a target for miR-128-3p and 142-3p; and nemo like kinase (NLK), a target for miR-3068-3p and 410-3p.

After determining differentially expressed rat serum EVs small RNAs after exercise, we performed a miRNA – mRNA interaction analysis to look for a biological function for these miRNAs. Using mirPath software, and KEGG signaling pathways union, 8 pathways were targeted by those 12 differentially expressed miRNAs (**Figure 3A**). In addition, looking for gene union, we found that 23 KEGG signaling pathways were statistically regulated by those 12 miRNAs (Supplementary Table S3).

From those 23 pathways, MAPK signaling (*p* = 5.944 × 10–11) was the one showing the largest number of targeted mRNAs. From 51 MAPK genes targeted by differentially expressed miRNAs after acute exercise, 5 genes were targeted by more than 1 miRNA. The most regulated genes from the MAPK pathway were: *platelet derived growth factor receptor beta* (PDGFRA), a target for miR-93-5p, 410-3p, and 128-3p; *MAP kinase-interacting serine/threonine kinase 2* (MKNK2), a target for miR-93-5p and 128-3p; *Nuclear factor of activated T-cells 3* (NFATE3), a target for miR-128-3p and 103-3p; *transforming growth factor beta receptor 1* (TGFBR1), a target for miR-128-3p and 142-3p; and *nemo like kinase* (NLK), a target for miR-3068-3p and 410-3p (**Figure 3B**).

## Discussion

It is well established that exercise is an important factor for human health, among other reasons, due to its positive effect on the metabolism. Understanding the molecular mechanisms underlying how exercise promotes health is a key factor in increasing exercise efficiency, and this understanding has great potential to unveil new strategies for metabolic disease treatment and early diagnosis. Here, we described the first exploratory study focusing on how acute exercise alters circulating EVs small RNAs. To our knowledge, this is also the first study describing *R. norvergicus* EVs small RNAs.

We find a slightly decrease in EVs modal diameter from high intensity exercise rats compared to other groups. Determining EVs size is important for the correct characterization of the vesicles present in a preparation. Some authors have described small vesicles derived from the endosomal pathway, called exosomes (40 to 100 nm), which are smaller in diameter compared to vesicles derived from outward budding of the plasma membrane, called microvesicles (up to 1 μm), or from vesicles derived from the activated apoptotic cell (Keam and Hutvagner, 2015). However, EVs diameters are very heterogeneous, depending on the source of purification. Cell culture supernatants contain more homogeneous vesicles compared to biological fluids, such as serum and plasma (Hu et al., 2010). Our purified serum EVs were in the range of 90 nm, suggesting that exosomes and small microvesicles are the greater contributors in our preparation.

Based on the small differences found in EVs modal diameter between moderate intensity exercise (91.5 nm) and high intensity exercise (85 nm) *p* = 0.028, we believe that the differences found may not have a biological effect, and other experiments should be done to confirm the decrease in EVs diameter after acute high intensity exercise. If it is confirmed, may suggest that high intensity exercise increases the release of smaller vesicles, such as exosomes.

Although Exoquick^TM^ can co-purify both high density and low density lipoproteins (HDLs and LDLs) present in serum (Keller et al., 2007), which can carry miRNAs (Kuhn et al., 2010), we didn’t see an increase in ApoA-IV protein marker after exercise, indicating that the contribution of lipoproteins in the transport of miRNAs in our preparation is more prevalent in non-exercised rats. Then, the contribution of lipoproteins in the transport of miRNAs seems to be decreasing after exercise, being diluted by CD63 positive vesicles.

We also demonstrated that acute aerobic exercise increases EVs concentration and EVs bearing CD63 protein.

Previously, plasma EVs from young men were described as showing that a single bout of cycling and running exercise could increase the levels of circulating EVs after exercise (Kyu et al., 2016), in agreement with our findings. The author suggested that increases in bloodstream EVs concentration after exercise could be related to the physiological activation state of the body during exercise. EVs could aid the disposal of cellular waste products generated under stress conditions and help the cells to maintain homeostasis, as well as playing roles in immune modulation, inflammatory responses activating tissue repair and vascular biology, including angiogenesis and cardioprotection (Kyu et al., 2016). Another study that is in agreement with our findings showed that exercise can increase levels of EVs in the heart and serum of diabetic mice after aerobic exercise (Lässer et al., 2012). We cannot state the origin of these secreted vesicles after exercise, but we can speculate from published data in the literature that the major contributing tissues to the release of EVs after exercise are muscle tissue (Laterza et al., 2009), endothelial cells (Lundberg et al., 2016) and immune system cells (Mach et al., 2016).

Cell culture experiments under normoxia and hypoxia conditions can elucidate the studies of EVs *in vivo* after acute exercise. In cell culture experiments, cardiomyocytes increased EVs release under hypoxia environment (MacPherson et al., 2015; Mi et al., 2015). Therefore, part of the augmented concentration of EVs in rat serum after exercise could be a consequence of the reduction in the oxygen supply at the tissue level.

After looking at the increase in serum concentration of EVs after acute aerobic exercise, we investigated the small RNAs profile associated with EVs. We used NextGene software to align mappable reads to RNA databases, and we identified 41 mature miRNAs with at least 5 reads, from 765 mature miRNAs of *R. norvegicus* described in miRBase. This number is important because it represents the diversity of miRNA transported by EVs, which is a small number compared to the concentration of intracellular miRNAs. However, these miRNAs are just a subset of serum circulating miRNAs. Results from two human studies looking for serum circulating miRNAs found more miRNAs in serum than our work (Mitchell et al., 2008; Neufer et al., 2015), since they did not isolate EVs, and serum contains EVs free miRNAs, miRNAs associated with EVs, and miRNAs associated with proteins and HDL∖LDL (Nielsen et al., 2014).

Scientific interest regarding circulating miRNAs and exercise was raised in 2009, when plasmatic circulating miRNAs were studied as a biomarker for tissue injury (Chaturvedi et al., 2015). Then, the interest in circulating miRNAs and exercise turned to the usage of miRNAs as a marker for maximal oxygen consumption. For example, in rowers, plasma levels of circulating miRNAs were verified after a 90-day training period. Plasma miRNAs followed specific profiles in response to (1) acute exercise only, (2) sustained exercise training only, and (3) acute and sustained exercise training. In addition, some of the plasma miRNAs (miR-146a and miR-20a) positively correlated with peak exercise capacity and cardiorespiratory fitness (Colombo et al., 2014). Our group demonstrated in a previous report that muscle specific miRNAs (miR-1, 133a and 206) increased in plasma immediately after a half marathon in five trained young male runners (Da Sol Kim et al., 2015). Another study focused on the impact of a single bout of high-intensity resistance exercise (RE) on skeletal muscle and circulatory miRNAs, simultaneously. From 30 selected miRNAs, 6 miRNAs were found altered within muscle tissue after exercise, while just 2 circulating miRNAs (miR-133a, miR-149) were found increased 4 h after exercise (Orton et al., 2005). In horses submitted to endurance riding, 167 miRNAs were found differentially expressed in whole blood when compared before and after the 160 km competition. Many of these miRNAs regulated genes involved in glucose metabolism, fatty acid oxidation, mitochondrion biogenesis, and immune response pathways (Palanisamy et al., 2010).

Aoi hypothesized that muscle-enriched miRNAs existing in circulation mediate beneficial metabolic responses induced by exercise. Cycling acute or chronic exercise did not change the serum levels of muscle-enriched miRNAs (miR-1, miR-133a, miR-133b, miR-206, miR-208b, miR-486, and miR-499) with an exception for miR-486, which showed a significant negative correlation with VO_2max_ (Pedersen et al., 2007).

In our results, we found out that rno-miR-128-3p, 103-3p, 148a-3p, 191a-5p, 93-5p, 25-3p, 142-5p, and 3068-3p decreased after exercise in rat serum EVs (**Table 1**). In humans, a miRNA profile from blood and plasma obtained from endurance and strength athletes, miR-128-3p showed correlation levels with the free testosterone (Pershing et al., 2015). miR-103-3p was studied in epicardial adipose tissue (EAT), which is important for obesity, intra-abdominal visceral fat, and insulin resistance. EAT thickness is a risk factor for cardiac diseases. It was shown that human miR-103-3p was suppressed in EAT of coronary artery disease patients is one of the possible mechanisms targeting a chemokine CCL3 in EAT (Pisitkun et al., 2004). Also, it was demonstrated that miR-103a-3p inhibited human adipose tissue-derived mesenchymal stem cell (hADSC) proliferation and osteogenic differentiation by binding to specific target sequences in the -CDK6 mRNA 3′-untranslated region (UTR) (Raposo and Stoorvogel, 2013). Altogether, this evidence makes miR-103a-3p a good candidate for further studies in the context of exercise metabolism.

**Table 1:** Statistical values of differentially expressed rat serum EVs miRNAs (DE-miRNA) and tRNAs (DE-tRNA) after low, moderate and high-intensity acute exercise.

	DE-miRNA							
	logFC	Low	Mod	High	logCPM	LR	PValue	FDR
rno-miR-128-3p	-0.14	-2.14	-3.00	-3.57	10.43	14.93	0.0001	0.0238
rno-miR-103-3p	-0.16	-2.34	-3.27	-3.89	8.12	13.10	0.0003	0.0284
rno-miR-330-5p	0.21	3.21	4.49	5.35	7.10	12.54	0.0004	0.0284
rno-miR-148a-3p	-0.11	-1.70	-2.38	-2.83	11.74	11.25	0.0008	0.0342
rno-miR-191a-5p	-0.10	-1.43	-2.00	-2.38	17.14	11.24	0.0008	0.0342
rno-miR-10b-5p	0.09	1.37	1.91	2.28	13.90	10.44	0.0012	0.0381
rno-miR-93-5p	-0.11	-1.70	-2.38	-2.83	7.96	10.17	0.0014	0.0381
rno-miR-25-3p	-0.10	-1.43	-2.00	-2.38	10.98	10.23	0.0014	0.0381
rno-miR-142-5p	-0.12	-1.86	-2.60	-3.10	10.07	9.81	0.0017	0.0414
rno-miR-3068-3p	-0.16	-2.42	-3.39	-4.03	7.04	9.32	0.0023	0.0454
rno-miR-142-3p	0.16	2.40	3.35	3.99	7.73	9.27	0.0023	0.0454
rno-miR-410-3p	0.18	2.68	3.76	4.47	7.07	9.08	0.0026	0.0461

	**DE-tRNA**							

chr7, trna8336-AspGTC	-1.13	-17.02	-23.83	-28.37	7.33	13.98	0.0002	0.0324

miR-25 was found decreased in a culture of smooth muscle cells from airways after being stimulated with pro-inflammatory cytokines, such as IL-1β, TNF-α, and IFN-g. miR-25 targets include KLF4, a protein with an important role in inflammation, proliferation, and differentiation of smooth muscle cells. KLF4 is a key regulator of monocyte differentiation and helps in the activation of proinflammatory genes in response to IFN-g, LPS or TNF-α (Robinson et al., 2010). We found miR-25 decreased after exercise, which could be related to the inflammatory state caused by acute aerobic exercise. In cardiac hypertrophy, miR-142 is a potent repressor of cytokine signaling in the myocardium (Rönn et al., 2014).

After exercise, four rat serum EVs miRNAs were found increased: rno-miR-330-5p, 10b-5p, 142-3p, and 4100-3p. It was demonstrated in Zebrafish embryo that miR-10a∖b had high expression in endothelial cells and regulated blood vessel outgrowth during development. miR-10a∖b target mib1 and Notch signaling. Inhibition of mib1 and Notch signaling partially rescued the angiogenic defects in miR-10 morphants, suggesting that angiogenic defects in miR-10a/10b morphants are caused by upregulation of Notch signaling (Safdar et al., 2016). Our results suggest that endothelial cells might be a powerful source of EVs delivering miR-10a/10b after exercise. miR-410-3p is known to directly target lactate dehydrogenase A (LDHA), a gene selectively repressed in normal insulin secreting b-cells. Human embryonic stem cells (hESCs) can be induced to express miR-410, keeping LDHA levels in check, which were then differentiated *in vitro* into pancreatic endoderm (Safdar and Tarnopolsky, 2017).

When we looked at the differentially expressed miRNAs target genes, we found that one of the main pathways involved with up or down regulated miRNAs was the MAPK signaling pathway. The MAPK pathway is composed of several proteins responsible for receiving a signal from the surface of the cell and for transducing the signal to the DNA in the nucleus. They act by activating or disabling proteins by the addition of phosphate groups to the neighboring proteins (Sawada et al., 2013). This pathway was described as inhibited in muscle biopsies from 10 moderately trained men submitted to aerobic and resistance exercise (Schwarzenbach et al., 2014). The same inhibition of MAPK was found in brains from mice fed a high-fat diet submitted to a single bout of exercise (Sharma et al., 2012). Also, the MAPK signaling pathway was down-regulated in adipose tissue after 6 months of aerobic exercise training (Sharp et al., 1985).

PiRNA biology is not fully understood. We found at least 10 piRNAs highly expressed in EVs, although none had its presence significantly affected by exercise. Their main known function is silencing transposable elements in germ line cells (Siomi et al., 2011), which has no direct relation with exercise. These piRNAs could be altered in other conditions. Regarding transfer RNA (tRNA), it is known as an adaptor molecule, which acts as a link between mRNA and amino acids sequence of proteins (Vacca et al., 2015). Here we described a tRNA, tRNA8336-AspGTC, downregulated after exercise in rat serum EVs. Further studies are necessary to understand the role of this tRNA in acute exercise adaptation. Besides their function as an adaptor molecule, tRNAs also serve as a major source of small non-coding RNAs that contain distinct and varied functions (Valadi et al., 2007). Recently, differences in codon usage preferences were reported as having a role in dependent tumorigenesis (Van Deun et al., 2014). Also, mutation of a tRNA gene that is specifically expressed in the central nervous system can cause neurodegeneration (Vickers et al., 2011) Specific tRNA is upregulated in human breast cancer cells as they gain metastatic activity, and experiments regarding gain-and-loss of function described two tRNAs, tRNAGluUUC and tRNAArgCCG, as promoters of breast cancer metastasis (Vlachos et al., 2015). There is compelling evidence that tRNA fragments, called tRFs, are involved in gene repression, inhibition of translation and cell proliferation (Valadi et al., 2007; Wang et al., 2012).

This descriptive study is important as a starting point in the understanding of the global pattern of serum EVs small RNAs after acute exercise. Altogether, we showed that acute aerobic exercise can increase rat serum EVs concentration, and that exercise could change the small RNA EVs profile. These small RNAs might contribute to the benefits of exercise to health and play roles in physiological adaptation to exercise. Further studies of these individual small RNAs need to be addressed to answer these questions.

## Materials and Methods

### Animals and Exercise Protocol

The experiments were carried out with 18 isogenic rats from the Wistar strain, which were submitted to 4 different conditions: as non-exercised (control, *n* = 4), as exercised with low intensity (*n* = 5), with moderate intensity (*n* = 4) and with high intensity (*n* = 5). We followed, with minor modifications, the protocol described for rat acute exercise on the treadmill described by Wang J. et al. (2016). All animals underwent an initial phase of acclimatization and adaptation before the exercise bout. The acclimatization took one 1 week, during which animals stayed 10–30 min per day on a non-moving treadmill. The adaptation also took one 1 week on a daily exercise session on the treadmill at 12 m.min^-1^ for 20 min. The exercise bout sessions lasted 40 min. The non-exercised group stayed 40 min on the non-moving treadmill. The low intensity exercised group was exercised 20% below the maximum lactate steady state (MLSS) at the speed of 14–16 m.min^-1^ on a treadmill. The moderate intensity exercised group was exercised at the speed of 20–22 m.min^-1^, on the MLSS. The high intensity exercised group was exercised at the speed of 24–26 m.min^-1^, 20% above the MLSS. The MLSS for Wistar rats was previously determined (Wang X. et al., 2016). Rats’ weight is shown in Supplementary Table S1. Animals were kept on cycles of 12:12 h of light and dark in rooms at 23 ± 2°C with *ad libitum* water and food. The Wistar strain was obtained from the animal facilities of the Federal University of São Paulo. The study was approved by the Animal Use Ethics Committee of the University of Brasília, Brazil. All procedures were performed according to the Brazilian College of Animal Experimentation.

### Blood Collection

Immediately after the end of a single exercise bout, rats were euthanized by lethal sedation with 2% xylazine (50 mg.kg^-1^) and 10% ketamine (80 mg.kg^-1^). After sedation, a cardiac puncture was performed to drain the blood. Tubes without anticoagulant were used to collect from 4 to 7 ml of blood. Platelet-depleted serum was obtained by centrifugation at 1,200 × *g* for 15 min up to 1 h after collection. The platelet-depleted serum was stored at -80°C.

### Extracellular Vesicles Purification

Extracellular vesicles purification was performed using the commercial kit Exoquick^TM^ (Exosome Precipitation Solution, System Biosciences Inc., Mountain View, CA, United States) following manufacturer’s recommendations. Initially, 500 μL of platelet-depleted serum was centrifuged at 3,000 × *g* for 15 min to ensure complete removal of cells and cellular debris. The supernatant was collected and 126 μL of Exoquick reagent was added. The mixture was incubated overnight at 4°C. Exoquick/serum mixture was centrifuged at 1,500 × *g* for 30 min at room temperature. The supernatant was discarded by aspiration, and the tubes were centrifuged at 1,500 × *g* for 5 min to ensure Exoquick removal. A volume of 200 μL of 1X PBS was used to resuspend the pellet. The EVs characterization was based on 50 μL, and the small RNA purification protocol was carried out using the remaining 150 μL.

### Immunoelectron Microscopy

Immunoelectron microscopy for rat serum EVs visualization was performed following the previously described protocol (Witwer, 2015). Briefly, a drop containing 10 μL of pooled EVs purified from all samples was placed on parafilm and processed for negative staining. Grids were washed three times in PBS 1X. The EVs were fixed in a drop of 2% paraformaldehyde for 10 min and washed in PBS prior to immunostaining. The grid was incubated in 30 μL of primary antibodies anti-CD63 (Santa Cruz Biotechnology, dilution 1:50) in PBS for 40 min, and washed with 0.1% BSA in PBS. Then, the grids were incubated with secondary anti-IgG rabbit antibody conjugated with 10 nm gold particle (Abcam, dilution 1:20). Grids were postfixed with 2.5% glutaraldehyde and incubated for 10 min and washed five times using distilled water. Finally, grids were contrasted with 3% uranyl acetate for 15 min. The images were acquired using 80 KVs in a transmission electron microscope JEOL model 1011, at the University of Brasilia electron microscopy laboratory.

### Estimating the Concentration and Size Distribution of EVs Using Tunable Resistive Pulse Sensing (RPS)

Serum EVs concentration and size distribution were estimated using Resistive Pulse Sensing technology (qNano, IZON, Christchurch, New Zealand). Briefly, the TRPS measures the electrical current flow passing through an adjustable pore. Single particles are measured in real time with high performance. EVs samples were diluted 40 times in PBS 1X buffer, filtered using 0.22 μm filters and measured by TRPS using NP150 pore size. The equipment was set to 49 mm pore stretch, 0.28 volts, and 1 cm.H_2_O pressure. The average current for measuring all samples ranged between 126.83 and 129.30 nA. The average diameter analysis, mode and particle concentration were performed using Izon Control Suite version 2.2 software. All samples were measured using beads of known concentration and diameters diluted in PBS.

### Identification of EVs Protein Markers by Western Blotting

Extracellular vesicles (EVs) protein purification was performed using aliquots of 5 μL of EVs suspension, diluted in 50 μL RIPA 1X protein lysis buffer (150 mM NaCl; 1.0% Triton X-100, 1% sodium deoxycholate, 0.1% SDS, 25 mM Tris-HCl, pH 7.4) plus 0.5 μL protease inhibitor cocktail (Sigma) and vortexed for 20 s. The total protein quantification was performed using the Protein Assay kit Qubit^®^ (Life Technologies) according to the manufacturer’s recommendations. Twenty (20) μg of total protein were separated by electrophoresis in 12% SDS-PAGE using Bio-Rad Mini-Protean II cast system. Electrophoresis was performed with 150 V and 15 mA for 2 h in parallel. One gel was stained with Coomassie blue and the other gel was transferred onto polyvinylidene difluoride membranes (PVDF, Bio-Rad). The transfer was performed using the Trans-Blot^®^ SD semi-Dry transfer cell (Bio-Rad) in transfer buffer containing 48 mM Tris, 39 mM glycine, 20% (v/v) methanol and 1.3 mM SDS for 1 h at 15 V and 200 mA. The membranes were blocked for 1 h in 3% BSA diluted in TBST 1X buffer (50 mM Tris.HCl pH 7.4; 150 mM NaCl; 0.1% Tween 20). After blocking, the membranes were incubated with rabbit primary antibody polyclonal anti-CD63, anti-ApoA-IV and anti-calnexin (Santa Cruz Biotechnology) at a concentration of 1: 500 diluted in 1% BSA in TBST buffer 1X and incubated overnight. Anti-IgG secondary antibody (1: 1000; Sigma) conjugated with alkaline phosphatase was applied to the membranes and incubated for 1 h in 1% BSA diluted in TBST buffer. Membranes were developed using developer NBT/BCIP (AP color development buffer, Bio-Rad) until the appearance of color. The reaction was stopped with distilled water for 10 min.

### EVs Small RNA Purification

The EVs small RNA purification was performed using miRCURY^TM^ RNA Isolation Kit (Exiqon) following the manufacturer’s recommendations. Briefly, 150 μl of EVs suspension was mixed with 60 μl of BF lysis solution, vortexed for 5 s and incubated for 3 min at room temperature. Then 20 μl of BF protein precipitation solution was added. The mixture was vortexed for 5 s, incubated for 1 min at room temperature and centrifuged for 3 min at 11,000 × *g*. The supernatant was transferred to a fresh tube, and 270 μl of 100% isopropanol was added. The mixture was vortexed for 5 s, and the sample was transferred to microRNA Mini Spin Column LF and incubated for 2 min at room temperature. The columns were centrifuged for 30 s at 11,000 × *g*. The columns were washed using 100 μl of 1BF washing solution and centrifugation for 30 s at 11,000 × *g*. The columns were washed using 700 μl of wash buffer 2BF in the column and centrifuged for 30 s at 11,000 × *g*. The last washing step was carried out adding 250 μl of wash buffer 2BF to the column and centrifuging for 2 min at 11,000 × *g*, aiming to guarantee the complete column drying. The RNA was eluted from the column after washing it twice with 50 μl of RNase-free water, incubation for 1 min at room temperature and centrifuging for 1 min at 11,000 × g. The 2100 Bioanalyzer (Agilent) small RNA chip was used to analyze concentration and size from the purified small RNA samples. This kit allows rapid and sensitive analysis of small nucleic acids from 6 to 150 nt in size.

### Statistical Analysis of the Data

The Shapiro–Wilk test was used to check data normality for EVs concentration, EVs proteins, and EVs small RNAs concentrations. Statistical comparisons were performed using nonparametric Kruskal–Wallis and Mann–Whitney *U* test for multiple comparisons. Values were considered statistically significant if *p* < 0.05.

### Sequencing of Small RNAs in Illumina GAII-X

The purified and characterized RNA samples (non-exercised *n* = 4, low exercised *n* = 5, moderate exercised *n* (4, high exercised n (5) were dried using RNAstable (Biomatrica). The small RNA library preparation (Truseq small RNA library preparation kit, Illumina), 36 bp single read sequencing (Genome Analyzer IIx, Illumina) were performed by Genomic Center at Catholic University of Brasilia (UCB, Brasília, Brazil).

### Small RNA Data Analysis

The sequencing results were processed using the commercial software NEXTGENE^®^ (SoftGenetics). The quality parameters used were: median score threshold ≥ 13 and *called base number of each read* ≥ 16. The software converted data files from FastQ to fasta. After filtering the low-quality reads and trimming the adapters, *R. norvegicus* sequencing databases were used to align the selected reads. The databases’ alignment procedures were applied in the following order: mature miRNAs available in miRBase (Rnor_5.0), piRNAs available in piRNAbank (rat database, Rnor_5.0), tRNA from GtRNAdb (Rnor_5.0) and rRNA available from Ensembl database (Rnor_5.0). Reads aligned to each database were removed to the following alignment.

The statistical analysis of read counts was carried on R (version 3.4.0) using edgeR A, B (version 3.18.1) (Yamamoto et al., 2015) and the heatmaps were generated with the pheatmap library. To determine which small RNAs had their expression influenced by exercise, the intensity was modeled according to the treadmill average speed (1, 15, 21, and 25 m.min^-1^) – we attributed 1 to the speed for non-exercised, to account for the fact they could still move. As recommended by the edgeR authors, transcripts with counts for only one or no samples were removed from the analysis. Data normalization factors were calculated by the weighted trimmed mean of *M*-values (“TMM”) method, and the generalized linear model (GLM) robust dispersion was estimated prior to fitting of binomial models. Differentially expressed transcripts were determined using a GLM likelihood ratio test. The *p*-value was adjusted for false discovery rate (FDR) using the Benjamin–Hochberg procedure, and small RNAs were considered statistically significant if FDR < 0.05.

### miRNAs – mRNAs Signaling Pathway Interaction

After describing 12 miRNAs differentially expressed after exercise in rat serum EVs, we performed a miRNAs – mRNAs signaling pathway interaction. Briefly, we used the online available software mirPath v.3 (Yu et al., 2012). We used the *R. norvegicus* database and the microT-CDS for mRNA target prediction. *P*-value and MicroT thresholds were maintained as default, 0.05 and 0.8, respectively. False discovery rate (FDR) correction was applied.

## Author Contributions

GO designed and performed the experiments. WP, CP, and NF contributed to bioinformatics data analysis. LP contributed to EVs purification, tunable resistive pulse sensing, and western blotting experiments. BP and JA designed and performed the rats training on the treadmill. JV contributed to western blotting. OF contributed to the design and implementation of the research, contributed to the final version of the manuscript. RP supervised the project, contributed to the analysis of the results and to the writing of the manuscript.

## Conflict of Interest Statement

The authors declare that the research was conducted in the absence of any commercial or financial relationships that could be construed as a potential conflict of interest.
