# Supplementary material for: Effects of Acute Aerobic Exercise on Rats Serum Extracellular Vesicles Diameter, Concentration and Small RNAs Content
Source: Front Physiol. 2018 May 24;9:532. doi: 10.3389/fphys.2018.00532 (PMC5976735; doi:10.3389/fphys.2018.00532)
Supplement: Supplementary file 1 [file Table_1.PDF]

Table S1. Wistar rats' weight used in the exercise protocol.

**Non-exercised (n=4)**	Weight (g)	**Low exercised (n=5)**	Weight (g)	**Moderate exercised (n=4)**	Weight (g)	**High exercised (n=5)**	Weight (g)
NE1	260	L1	310	M1	300	H1	270
NE2	290	L2	270	M2	265	H2	255
NE3	265	L3	300	M3	265	H3	300
NE4	275	L4	280	M4	330	H4	313
		L5	315			H5	285
**Average**	**272,5**	**Average**	**295**	**Average**	**290**	**Average**	**284,6**
**Std±**	**13,2**	**Std±**	**18,3**	**Std±**	**29,0**	**Std±**	**21,8**
