# Supplementary material for: Effects of Acute Aerobic Exercise on Rats Serum Extracellular Vesicles Diameter, Concentration and Small RNAs Content
Source: Front Physiol. 2018 May 24;9:532. doi: 10.3389/fphys.2018.00532 (PMC5976735; doi:10.3389/fphys.2018.00532)
Supplement: Supplementary file 2 [file Table_2.PDF]

Table S2. Raw reads, reads after trimming and alignment against miRNA, piRNA, rRNA, and tRNA *Rattus norvegicus* databases.

Samples	Total reads per sample	Trimmed reads	% reads after trimming	miRNA aligned reads	piRNA aligned reads	rRNA aligned reads	tRNA aligned reads
**NE1**	2371359	401022	16,91	26060	100452	2153	31297
**NE2**	395005	42852	10,85	3989	7821	526	2902
**NE3**	1530531	101331	6,62	9947	12744	398	3532
**NE4**	3700628	1069987	28,91	83937	140111	33632	83845
**L1**	281733	68718	24,39	8009	6802	1330	4856
**L2**	1741490	153059	8,79	7586	15826	947	7134
**L3**	362405	68326	18,85	4984	10899	686	3992
**L4**	1350427	607711	45,00	127029	129338	7270	36210
**L5**	1037888	334571	32,24	51409	58979	5279	24239
**M1**	364047	37659	10,34	2145	4728	261	2867
**M2**	112088	31653	28,24	1860	2380	287	7550
**M3**	3131297	459000	14,66	23404	34220	9332	25005
**M4**	1916749	570477	29,76	24970	67328	13179	39156
**H1**	3484966	2078111	59,63	91458	429218	37449	110623
**H2**	2200154	371520	16,89	9019	56069	3394	18431
**H3**	931756	169363	18,18	5185	22261	1191	9923
**H4**	1623795	88560	5,45	3406	7325	1052	8240
**H5**	240910	41655	17,29	1423	7691	548	6669
**Total**	26884765	6699820	24,92	485830	1114211	118916	426481
