# Supplementary material for: Effects of Acute Aerobic Exercise on Rats Serum Extracellular Vesicles Diameter, Concentration and Small RNAs Content
Source: Front Physiol. 2018 May 24;9:532. doi: 10.3389/fphys.2018.00532 (PMC5976735; doi:10.3389/fphys.2018.00532)
Supplement: Supplementary file 3 [file Table_3.PDF]

Table S3. Rat serum EV miRNAs involved in KEGG pathways.

KEGG pathway	p-value	#genes	#miRNAs
MAPK signaling pathway	5.94E-11	51	10
Transcriptional misregulation in cancer	2.01E-07	32	10
MicroRNAs in cancer	2.93E-05	27	9
Renal cell carcinoma	5.03E-05	16	6
FoxO signaling pathway	6.29E-05	25	10
Pathways in cancer	0.000138281	48	9
Mucin type O-Glycan biosynthesis	0.000385403	5	3
PI3K-Akt signaling pathway	0.000421404	47	9
Circadian rhythm	0.001189654	9	6
Proteoglycans in cancer	0.001682613	33	8
Chronic myeloid leukemia	0.001682613	16	8
Ras signaling pathway	0.009829894	32	9
Estrogen signaling pathway	0.016231474	13	5
N-Glycan biosynthesis	0.016231474	7	8
Protein processing in endoplasmic reticulum	0.016231474	25	8
Neurotrophin signaling pathway	0.021299765	21	7
Amphetamine addiction	0.021902601	10	6
Prostate cancer	0.024691048	15	7
Cocaine addiction	0.027692342	8	7
Vasopressin-regulated water reabsorption	0.037500176	10	7
Cell cycle	0.037500176	21	8
Regulation of actin cytoskeleton	0.037500176	28	9
mTOR signaling pathway	0.048088242	11	6
