# Supplementary material for: Effects of Acute Aerobic Exercise on Rats Serum Extracellular Vesicles Diameter, Concentration and Small RNAs Content
Source: Front Physiol. 2018 May 24;9:532. doi: 10.3389/fphys.2018.00532 (PMC5976735; doi:10.3389/fphys.2018.00532)
Supplement: Supplementary file 4 [file Image_1.PDF]

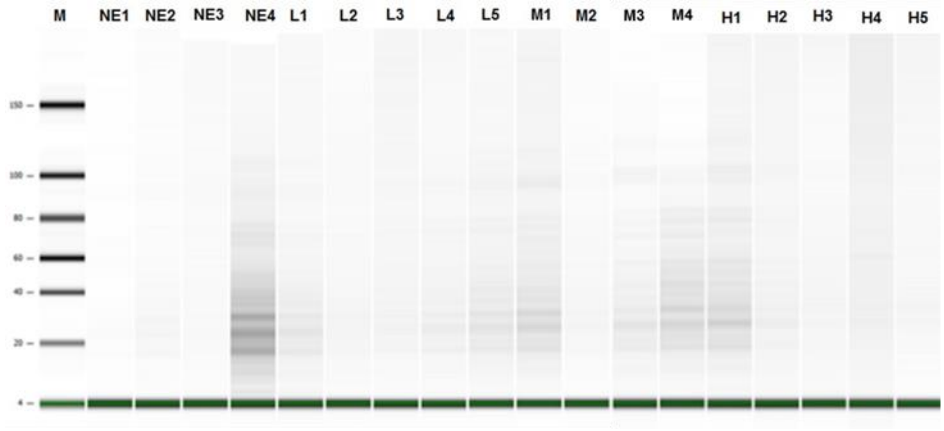

Figure S1. Small RNA characterization purified from rat serum EVs. All samples show RNA ranging from 20 to 60nt in size and absence of larger RNAs. M=Marker; NE = non-exercised group; L= low intensity exercised group; M=moderate intensity exercised group; H= High intensity exercised group.
