# Supplementary material for: Effects of Acute Aerobic Exercise on Rats Serum Extracellular Vesicles Diameter, Concentration and Small RNAs Content
Source: Front Physiol. 2018 May 24;9:532. doi: 10.3389/fphys.2018.00532 (PMC5976735; doi:10.3389/fphys.2018.00532)
Supplement: Supplementary file 5 [file Image_2.PDF]

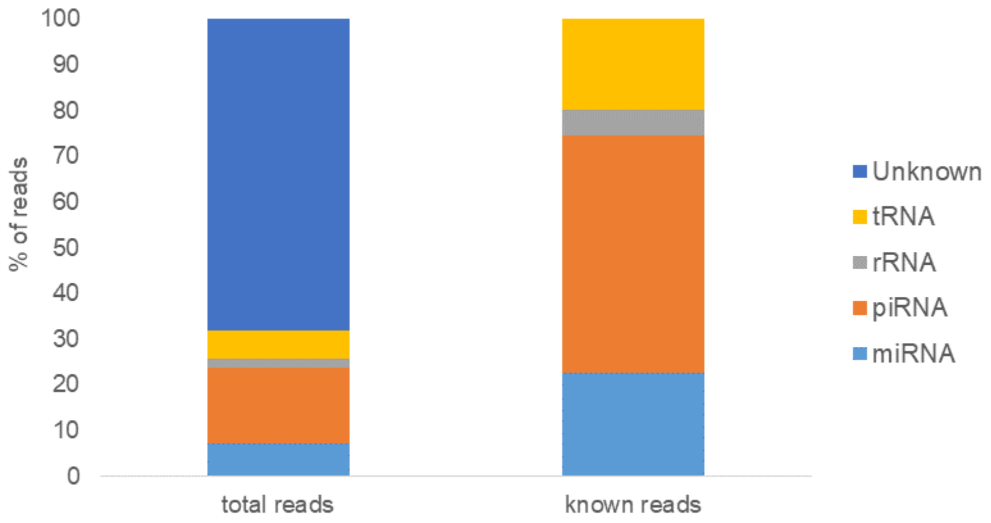

Figure S2. Percentage of mappable reads after sequencing of small RNAs from rat serum EVs. The first column represents the general profile of reads. The second column represents only the known reads mapped against *Rattus norvegicus* databases.
